# Surviving in a toxic world: transcriptomics and gene expression profiling in response to environmental pollution in the critically endangered European eel

**DOI:** 10.1186/1471-2164-13-507

**Published:** 2012-09-25

**Authors:** Jose Martin Pujolar, Ilaria AM Marino, Massimo Milan, Alessandro Coppe, Gregory E Maes, Fabrizio Capoccioni, Eleonora Ciccotti, Lieven Bervoets, Adrian Covaci, Claude Belpaire, Gordon Cramb, Tomaso Patarnello, Luca Bargelloni, Stefania Bortoluzzi, Lorenzo Zane

**Affiliations:** 1 Department of Biology, University of Padova, I-35131, Padova, Italy; 2 Department of Comparative Biomedicine and Food Science, University of Padova, I-35020, Legnaro, Italy; 3 Laboratory of Biodiversity and Evolutionary Genomics, Katholieke Universiteit Leuven (KU Leuven), B-3000, Leuven, Belgium; 4 Department of Biology, Università Roma Tor Vergata, I-00133, Rome, Italy; 5 Department of Biology, University of Antwerp, B-2020, Antwerpen, Belgium; 6 Toxicological Center, University of Antwerp, B-2610, Wilrijk, Belgium; 7 Research Institute for Nature and Forest, B-1560, Groenendaal-Hoeilaart, Belgium; 8 School of Medicine, University of St. Andrews, KY16 9TF, Fife, United Kingdom

**Keywords:** *Anguilla anguilla*, European eel, Transcriptome, Microarrays, Pollution

## Abstract

**Background:**

Genomic and transcriptomic approaches have the potential for unveiling the genome-wide response to environmental perturbations. The abundance of the catadromous European eel (*Anguilla anguilla*) stock has been declining since the 1980s probably due to a combination of anthropogenic and climatic factors. In this paper, we explore the transcriptomic dynamics between individuals from high (river Tiber, Italy) and low pollution (lake Bolsena, Italy) environments, which were measured for 36 PCBs, several organochlorine pesticides and brominated flame retardants and nine metals.

**Results:**

To this end, we first (i) updated the European eel transcriptome using deep sequencing data with a total of 640,040 reads assembled into 44,896 contigs (Eeelbase release 2.0), and (ii) developed a transcriptomic platform for global gene expression profiling in the critically endangered European eel of about 15,000 annotated contigs, which was applied to detect differentially expressed genes between polluted sites. Several detoxification genes related to metabolism of pollutants were upregulated in the highly polluted site, including genes that take part in phase I of the xenobiotic metabolism (CYP3A), phase II (glutathione-S-transferase) and oxidative stress (glutathione peroxidase). In addition, key genes in the mitochondrial respiratory chain and oxidative phosphorylation were down-regulated at the Tiber site relative to the Bolsena site.

**Conclusions:**

Together with the induced high expression of detoxification genes, the suggested lowered expression of genes supposedly involved in metabolism suggests that pollution may also be associated with decreased respiratory and energy production.

## Background

The incorporation of genomic and transcriptomic approaches into ecological and evolutionary studies enables to further explore how natural populations respond to environmental change and anthropogenic pressures [1-4]. Genome-scale approaches have the potential for identifying genes and genetic networks that underlie ecologically important traits, such as those linked to adaptation to cold temperatures [5], defense mechanisms [6] or response to pollutants [7,8]. Changes in gene expression can be linked to phenotypic and environmental variation, hence advancing our understanding of the adaptive importance of gene functions and their ecological and evolutionary consequences [9]. The resilience of a species depends on its vulnerability in the face of environmental changes, which is determined by genetic composition and physiological tolerance [10]. Changes in environmental conditions can rapidly lead to phenotypic change, involving either genetic (local adaptation) or plastic (physiological plasticity) changes. In the well-established model species *Fundulus heteroclitus*, the species has evolved both highly plastic and locally adapted phenotypes within different selective contexts [11]. While plasticity has allowed the species to cope with environment variability, some *F. heteroclitus* populations have evolved pollution tolerance allowing them to survive concentrations of organic contaminants that are lethal to populations from clean habitats [12], in a response that is not plastic but adaptive and heritable [11].

While the evolutionary consequences of pollutants have been well explored in natural populations of freshwater fish using genomic and transcriptomic approaches (reviewed in [8]), less attention has been paid to diadromous fish species that complete their life-cycle through ontogenic shifts between freshwater and marine habitats (e.g. salmonids, anguillids). The study of diadromous species is particularly relevant as they represent a natural model to understand the combined impact of continental anthropogenic and oceanic climate stressors. Unlike other model fish species, very little is known about the ecological and evolutionary consequences in species with a catadromous life-strategy, such as the European eel *Anguilla anguilla*, which illustrates an example of a fish species designated by the IUCN as “critically endangered” and potentially strongly affected by human stressors throughout its life cycle.

The European eel is a facultative catadromous species with a particularly complex life cycle that includes an oceanic and a continental phase. After spawning in the Sargasso Sea, larvae cross the Atlantic Ocean following the prevailing currents and metamorphose into glass eels upon reaching the edge of the continental shelf off Europe and North Africa. Glass eels complete the migration into riverine, estuarine and coastal feeding habitats as yellow eels, and after a highly variable feeding period, they metamorphose into silver eels that migrate back to the Sargasso Sea utilizing their high fat reserves, where they reproduce once and die [13]. Homing to a single spawning ground explains the lack of genetic differentiation between geographic areas across Europe, suggestive of a panmictic population [14-16], while a large variance in parental reproductive success associated with fluctuating ocean conditions explains the pattern of genetic patchiness (significant genetic differences among samples lacking a clear geographical trend or pattern) found at local scale [17,18].

There has been a continuous decline of continental yellow eel recruitment since the 1950s and marked decadal reductions in glass eel recruitment since the early 1980s [19]. Over the last 5 years glass eel recruitment has been exceptionally low, averaging between less than 1% (continental North Sea) and 5% (elsewhere in Europe) of the 1960–1979 levels [19]. Several causes have been put forward to explain the decline of the eel stock including anthropogenic factors such as overfishing, pollution, habitat degradation and man-introduced parasites and diseases [13], as well as environmental factors such as climate and ocean current change [20-22]. Recent genetic data suggest that the demographic decline did not entail a genetic decline of the same magnitude, as evidenced by the observation of a moderate to high level of genetic diversity, no signatures of a bottleneck episode and comparable values of short- and long-term effective population size [23].

While eels are regarded as fairly resilient, often inhabiting unproductive waters and polluted habitats, they are prone to bioaccumulation of lipophilic contaminants due to their particular ecology (benthic feeding) and physiology (high fat content) [24]. Evidence has been presented that different kinds of chemical compounds such as polychlorinated biphenyls (PCBs), pesticides and toxic metals could have a serious impact on the health of the European eel [for a review see [25,26]. Maes et al. [27] observed a significant negative correlation between heavy metal pollution load and condition, as well as a reduced genetic variability in highly polluted eels originating from three Belgian river basins. It has been hypothesized that the accumulation of contaminants in the fat tissue of eels during the feeding stage could impair the quality of spawners [13,28]. In this sense, a considerable decrease in condition and lipid energy stores might be responsible for migrating adults failing to swim the 6,000 km distance to the Sargasso Sea [29], while mobilization of lipids and lipophilic contaminants to the gonads during the transoceanic migration might compromise production of good quality eggs and normal development of early larval stages [30].

Understanding the impact of pollutants at the genomic and transcriptomic level is a critical point in order to safeguard the evolutionary adaptive potential of the European eel. Several experimental studies on gene expression have shown genetic responses to pollutants in eel [31-36]. However, large-scale transcriptomic approaches in the European eel have been limited to the study of osmoregulation [37] and comparative gene expression analysis between sympatric European and American eel larvae in the Sargasso Sea [38]. Using the same microarray comprising about 6,000 anonymous probes, Kalujnaia et al. [37] identified 229 differentially expressed genes with a putative role in migration between freshwater and marine environments, while Bernatchez et al. [38] identified 146 genes showing different timing of expression between the two Atlantic eels. In this paper we first describe the development of an eel-specific microarray of about 15,000 annotated cDNAs based on a large collection of existing and novel high-throughput transcriptomic sequences. Our approach aims at identifying genes involved in the response to pollutants by means of a comparative analysis of gene expression between environments with a varying level of pollution. As such, we aim at assessing the potential fitness consequences of bioaccumulation levels in individual eels and pinpoint the metabolic pathways most influenced by such stressors.

## Results

### Transcriptome assembly and analysis

A total of 640,040 reads were assembled into contigs using MIRA 3 (Table 1). A first run of assembly produced 61,838 contigs with a mean length of 460.4 bp, ranging from 40 to 2,590 bp. On average, transcripts included 10.4 reads 181 (from 1 to 2,611, median value of 4), with a read cover-182 age of 5.3 (from 1 to 945.4, median value of 3.1). A second run conducted using the previously obtained contigs as input produced 52,125 contigs. Average length was similar to the first run (466.5 bp), but average quality increased from a mean of 45.1 (about 1 in 32,000 bp error rate) to 46.1 (about 1 in 41,000 bp error rate). A further quality check was conducted for the final set of putative transcripts by filtering sequences by length (minimum 200 bp) and average sequence quality (minimum of 30). A total of 44,896 contigs were obtained, with a mean length of 511 bp and an average sequence quality of 47.9 (corresponding to an average error rate of 1 in 62,000 bp).

**Figure 1:**
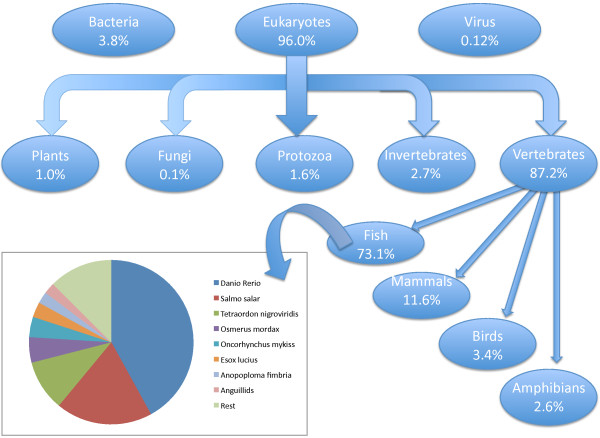
Relative abundance of European eel BLAST hits in main taxonomic groups using a simplified Tree of Life diagram.

Among all transcripts, 14,574 (28%) showed BLAST matches. Eukaryotes accounted for 96% of all BLAST hits, with vertebrates accounting for most (87.2%) of the hits (Figure 1). Among vertebrates, fish represented 73.1% of all hits, followed by mammals (11.6%). The most represented fish species was zebrafish *Danio rerio* (42.3%), followed by salmon *Salmo salar* (19.6%) and pufferfish *Takigufu rubripes* (10%). Anguillids represented less than 5% of hits among fish, including Japanese eel *A. japonica* (2%), European eel *A. anguilla* (1.6%) and American eel *A. rostrata* (0.3%).

**Table 1:** Statistics describing the distributions of different properties of contig sequences (a) obtained by the first run of assembly, (b) second run of assembly and (c) final set after filtering for minimum sequence length and average quality

**640,040 reads**		**Min**	**1st Q**	**Median**	**Mean**	**3rd Q**	**Max**
(a) 61,838 contigs	Length	40	301	413	460.4	551	2590
	Number of reads	1	2	4	10.4	10	2611
	Average coverage	1	1.9	3.1	5.3	5.7	945.4
	Average quality	14.8	34.9	39.4	45.1	55.2	88.7
	GC content	16.1	37.1	41.9	42.4	47.3	73.9
(b) 52,125 contigs	Length	40	299	413	466.5	564	2590
	Average quality	14.8	35	39.8	46.1	56.7	90
	GC content	16.1	37.1	41.8	42.3	47.2	73.9
(c) 44,896 contigs	Length	200	348	441	511	606	2590
after filtering	Average quality	30	36	41.6	47.9	58.6	90
	GC content	22	37.3	41.9	42.4	47.1	68.9

**Figure 2:**
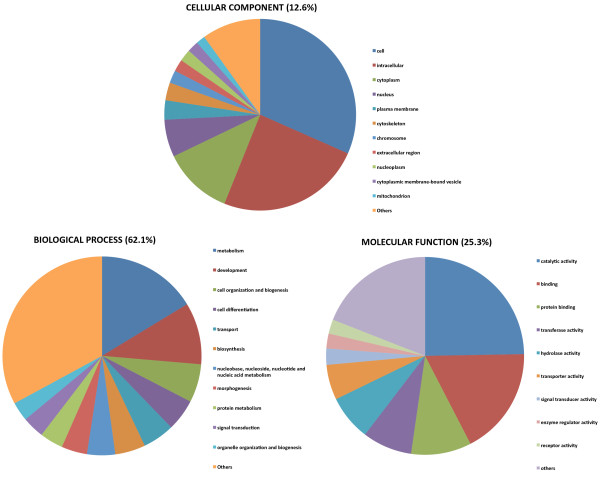
**Functional annotation of European eel contigs associated to GO terms.** GO classes were grouped into 112 GO-slim terms for biological process (BP), molecular function (MF) and cellular component (CC) ontologies.

Functional annotation using the Blast2Go suite showed 10,920 of the contigs (20.95%) associated to GO terms, for a total of 76,158 term occurrences. Using the webtool CateGOrizer, GO classes were grouped in a total of 112 Go-Slim terms including biological process (62.1%), molecular function (25.3%) and cellular component (12.6%) ontologies (Figure 2). Catalytic activity and binding represented the majority of molecular functions. Key processes such as metabolism and development were present among biological processes.

All contig sequences are available at Eeelbase release 2.0, a dedicated database implemented using the MySQL and Django web framework and freely available at: http://compgen.bio.unipd.it/eeelbase. The database is queryable by keywords and BLAST using nucleotide or protein sequences. For each contig (identified by Eeelbase ID) the FASTA sequence is provided together with the list of all reads belonging to the contig, BLAST results and associated GO Terms. The web site also allows to perform alignments with reference genomes using *on the fly* BLAT search against the latest releases of the zebrafish and stickleback genomes in the UCSC Genome Browser.

### Pollution analysis

Comparison of pollutants between the Tiber (N = 30) and Bolsena (N = 6) sites pointed to strong differences in pollution load between sites, with concentration of all pollutants measured being consistently higher in the tissues of silver male eels at the Tiber site than in those from the Bolsena site (Table 2), despite a high individual variability. Concentrations of PCBs were 6–7 times higher at the Tiber site but not significantly so using univariate ANOVA. The sum of the 7 main PCB congeners (IUPAC No. 28, 52, 101, 118, 138, 153 and 180) in tissue collected at the Tiber site (214.4 ng/g ww) was also above the Italian limit for consumption (75 ng/g ww), while at the Bolsena site this value was below the limit (37.6 ng/g ww). The value of Sum 7 PCBs at Tiber was comparable to the value reported in Miniero et al. [39] on eels collected from the urban tract of the river Tiber in Rome (167–372 ng/g ww). Organochlorine pesticides (OCPs) were twofold higher in the tissues of silver male eels at the Tiber site with significant differences for pp-DDE (p = 0.038) and pp-DDT (p = 0.007). Brominated flame retardants (BFRs) were 6 to 8 times higher at the Tiber site than at the Bolsena site, with both polybrominated diphenyl ethers (PBDEs) and hexabromocyclododecane (HBCD) isomers being highly significant (p < 0.001). The tissues collected at the river Tiber site also exhibited the highest concentrations for all nine metals measured but differences were not statistically significant due to the high individual variability. However, a multivariate ANOVA conducted on a set of all pollutants combined (Sum 36 PCBs, HCB, pp-DDE, pp-DDT, Sum 10 PBDEs, Sum 3 HBCDs and nine metals) indicated statistically significant differences (F = 2.7; df = 20; p = 0.022).

**Table 2:** Average concentration of pollutants in European eel silver males from the Tiber (N = 30) and Bolsena (N = 6) sites

	**Tiber**				**Bolsena**				
	**Mean**	**Min**	**Max**	**SD**	**Mean**	**Min**	**Max**	**SD**	**p**
**Sum 36 PCBs**	489.08	64.19	3836.97	650.59	73.63	14.68	245.01	86.99	0.132
**Sum 7 PCBs**	214.38	27.24	1442.19	242.09	37.63	6.01	120.32	43.00	0.087
**HCB**	6.99	2.60	13.99	2.31	4.35	1.35	16.76	6.12	0.071
**pp-DDE**	45.33	15.10	84.46	16.85	29.29	12.21	54.20	15.14	0.038*
**pp-DDT**	12.97	4.03	27.11	6.02	5.45	0.75	14.36	5.15	0.007*
**Sum 10 PBDEs**	44.00	11.42	86.49	18.31	6.68	1.54	25.82	9.43	<0.001*
**Sum 3 HBCDs**	53.16	3.03	101.63	21.93	6.42	0.55	31.72	12.42	<0.001*
**Ag**	0.021	DL	0.047	0.012	0.011	0.006	0.017	0.005	0.320
**As**	0.393	0.161	0.920	0.153	0.353	0.244	0.572	0.116	0.550
**Cd**	0.026	DL	0.352	0.074	DL	DL	DL	DL	0.401
**Co**	0.050	0.007	0.274	0.049	0.018	0.006	0.030	0.008	0.124
**Cr**	0.129	0.033	0.394	0.088	0.095	0.047	0.229	0.068	0.379
**Cu**	1.742	0.71	4.08	0.681	1.564	1.08	2.54	0.517	0.550
**Ni**	0.040	0.006	0.147	0.039	0.028	0.016	0.057	0.015	0.467
**Pb**	0.092	0.006	0.430	0.084	0.049	0.017	0.087	0.023	0.227
**Zn**	66.135	27.1	155.0	25.857	59.393	41.0	96.4	19.617	0.551
**(All Pollutants)**							F = 2.7	df = 20	0.022*

Univariate and multivariate ANOVAs for equal pollutant bioaccumulation are presented. A Multivariate ANOVA was also conducted on a set of all pollutants combined (Sum 36 PCBs, HCB, pp-DDE, pp-DDT, Sum 10 PBDEs, Sum 3 HBCDs and nine metals). Concentration of PCBs, OCPs and BFRs is expressed in ng/g of wet weight. Metal concentration is expressed in μg/g of dry weight. DL = below detection limit. * = statistically significant.

### Gene expression analysis

A European eel-specific microarray of 14,913 cDNAs was generated and used to identify genes involved in response to pollutants by comparing the expression profiles of 23 individuals from heavily-polluted (Tiber, 8 males) and lowly-polluted (Bolsena, 7 males and 8 females) environments. Mean concentrations of pollutants were consistently higher at the Tiber site, but not all individual measurements. Across all 23 microarray experiments, only 99 probes (0.6%) did not show a signal higher than the background, while 14,274 (95.7%) were successful (flag value equal to 1) in at least 16 out of 23 experiments.

**Figure 3:**
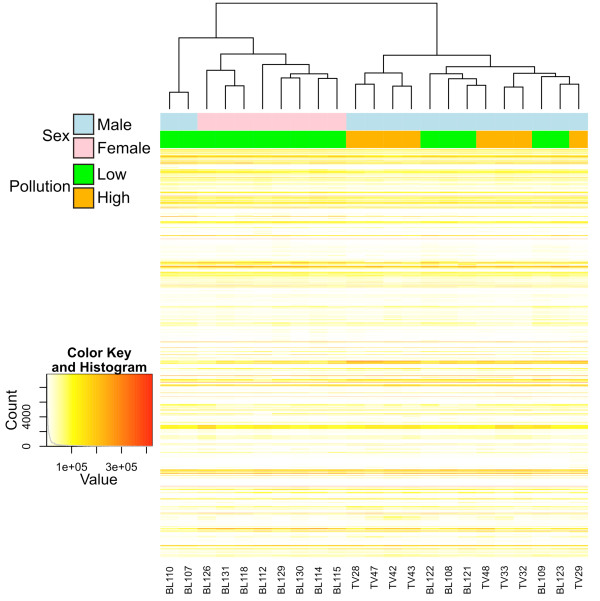
**Pearson-correlation based heatmap using the 2,000 genes with higher expression and variability across samples.** Individuals are colour-coded according to sex (pink, females; blue, males) and pollution (green, low; orange, high).

Prior to the analysis of differentially expressed genes, a Pearson correlation-based heatmap using a subset of 2,000 genes only partially grouped individuals according to sampling site (Figure 3), which is suggestive of similar environmental conditions. All females were from the same site (Bolsena) and clustered together, which is scarcely informative. However, males and females were well separated. Since differences in the expression of sex-linked genes could interfere with the identification of genes involved in response to pollutants, two separate analyses were conducted in parallel (1) considering only males and (2) with both males and females included.

A two-unpaired class Significance Analysis of Microarray (SAM) test was conducted on normalized data between individuals from the Tiber (N = 8 males) and Bolsena (N = 7 males) sites. Enforcing a stringent 0% false discovery rate (FDR), a total of 168 transcripts were differentially expressed in the samples from Tiber in comparison with the samples from Bolsena, 30 upregulated and 138 downregulated. With a less restrictive 5% FDR, a list of 1,171 probes were differentially expressed in the Tiber individuals in comparison with the Bolsena individuals, 406 over-expressed and 775 under-expressed.

**Figure 4:**
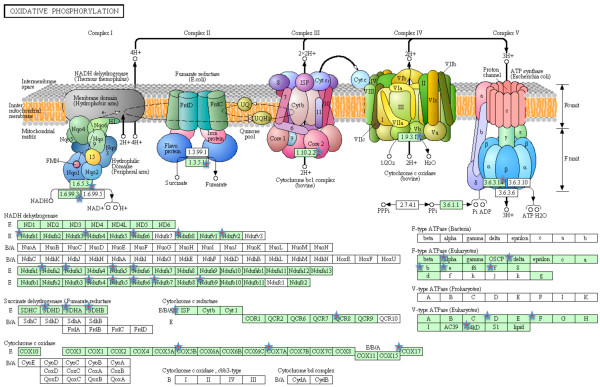
**Mapping of zebrafish genes homologous to European eel transcripts to oxidative phosphorylation.** Green boxes represent KEGG nodes specific to the considered organism. Red stars indicate enriched nodes, which may represent one or more genes.

**Figure 5:**
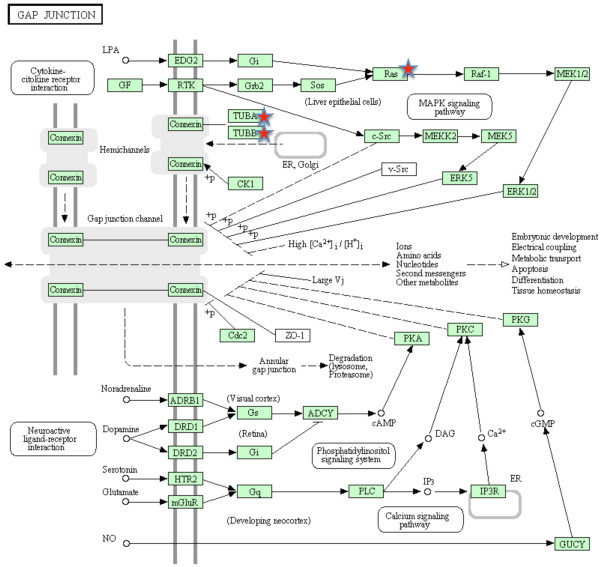
**Mapping of zebrafish genes homologous to European eel transcripts to gap junction.** Green boxes represent KEGG nodes specific to the considered organism. Red stars indicate enriched nodes, which may represent one or more genes.

**Figure 6:**
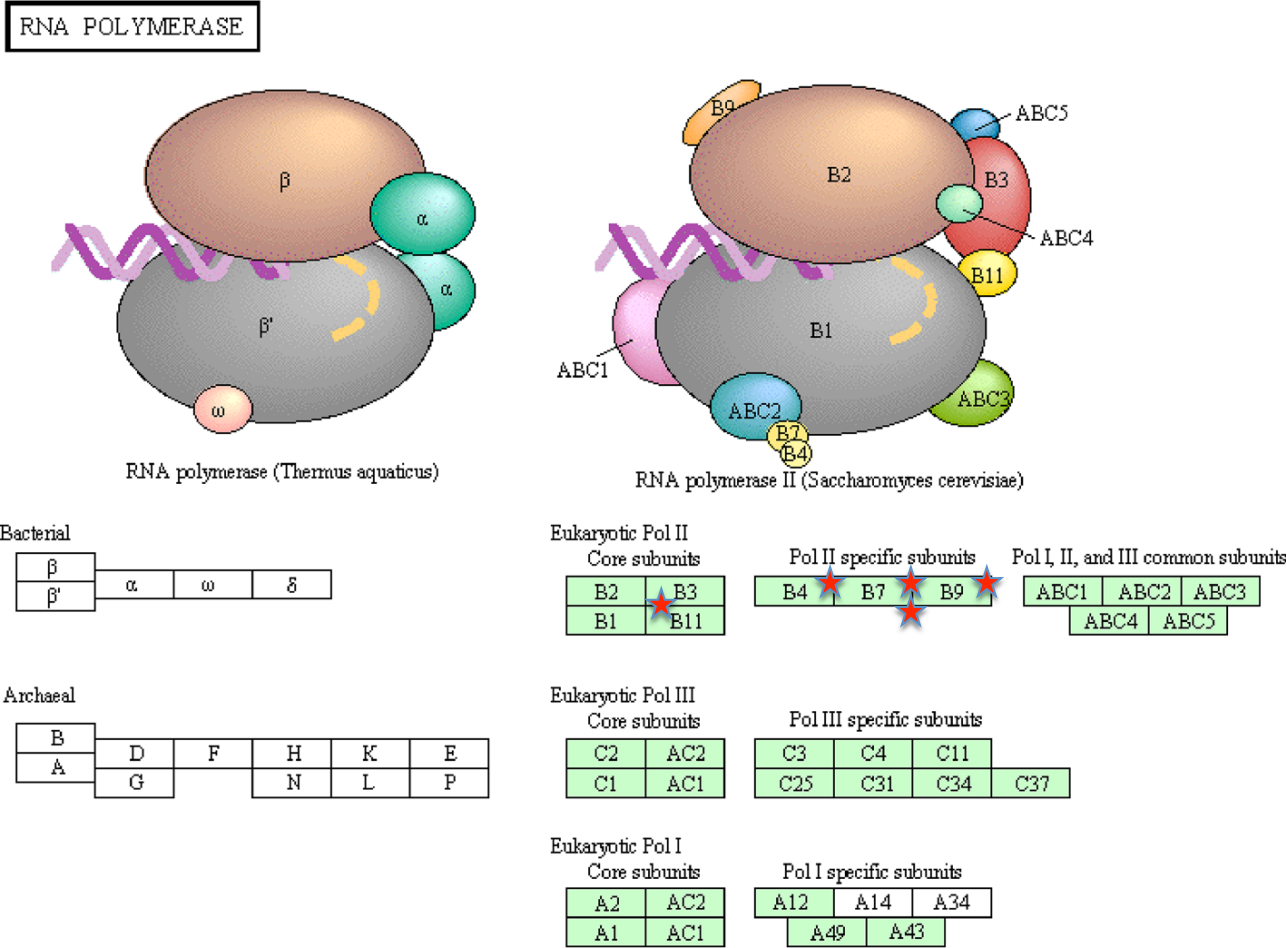
**Mapping of zebrafish genes homologous to European eel transcripts to RNA polymerase.** Green boxes represent KEGG nodes specific to the considered organism. Red stars indicate enriched nodes, which may represent one or more genes.

A more systematic functional interpretation of the set of differentially expressed genes between sites was obtained by an enrichment analysis in DAVID. Using the list of transcripts obtained with the 5% FDR corresponding to 709 unique transcripts, a putative match with zebrafish was found for 563 transcripts. Enrichment analysis showed a total of 23 terms, all significantly over-represented in the heavily-polluted Tiber site compared to the Bolsena site (Table 3), including 7 KEGG pathway terms, 5 MF-GO terms, 3 BP-GO terms, 3 SP-PIR (a database of protein superfamilies) keywords and 5 InterPro (a database of protein families). The genes assigned to the above 23 terms are mostly known to be involved in drug metabolism (Table 4). Those included genes encoding several members of the cytochrome P450 superfamily of enzymes (CYP2J23, CYP3A65, CYP46A1) that catalyze the oxidation of xenobiotic substances such as pollutants, drugs and toxic elements. A higher number of terms were under-expressed in the individuals from the Tiber site in comparison with the individuals from the Bolsena site (Table 5). The assigned genes are mostly known to be involved in energetic metabolism (Table 6). Three KEGG pathways were significantly enriched, namely oxidative phosphorylation (32 genes; Figure 4), gap junction (8 genes; Figure 5) and RNA polymerase (5 genes; Figure 6).

**Table 3:** Functional Annotation Enrichment analysis of statistically-significant over-expressed genes in European eel silver males from the river Tiber site (N = 8) in comparison with silver males from the lake Bolsena site (N = 7). FE = Fold Enrichment

**Category**	**Term**	**Count**	**P value**	**FE**
KEGG_PATHWAY	dre00052:Galactose metabolism	4	0.004	11.5
	dre00520:Amino sugar and nucleotide sugar metabolism	5	0.004	7.2
	dre00591:Linoleic acid metabolism	3	0.010	17.3
	dre00040:Pentose and glucuronate interconversions	3	0.016	13.8
	dre00511:Other glycan degradation	3	0.024	11.5
	dre00500:Starch and sucrose metabolism	3	0.042	8.7
	dre00980:Metabolism of xenobiotics by cytochrome P450	3	0.042	8.7
INTERPRO	IPR002401:Cytochrome P450, E-class, group I	4	0.007	9.5
	IPR017973:Cytochrome P450, C-terminal region	4	0.009	8.8
	IPR017972:Cytochrome P450, conserved site	4	0.009	8.8
	IPR001128:Cytochrome P450	4	0.011	8.1
	IPR012335:Thioredoxin fold	5	0.041	3.7
SP_PIR_KEYWORDS	heme	5	0.010	5.6
	monooxygenase	4	0.010	8.4
	oxidoreductase	8	0.023	2.7
GOTERM_BP	GO:0055114 ~ oxidation reduction	11	0.005	2.7
	GO:0019882 ~ antigen processing and presentation	4	0.007	9.6
	GO:0006955 ~ immune response	4	0.042	5.0
GOTERM_MF	GO:0009055 ~ electron carrier activity	7	0.013	3.5
	GO:0020037 ~ heme binding	5	0.025	4.3
	GO:0043167 ~ ion binding	27	0.037	1.4
	GO:0043169 ~ cation binding	27	0.037	1.4
	GO:0046906 ~ tetrapyrrole binding	5	0.039	3.8

**Table 4:** Functional Annotation Clustering of statistically-significant over-expressed genes in European eel silver males from the river Tiber site (N = 8) in comparison with silver males from the lake Bolsena site (N = 7)

**Annotation Cluster**	**Gene**
Annotation Cluster 1	Cytochrome c oxidase subunit 1
(Enrichment Score: 1.81)	Cytochrome P450, family 2 subfamily J, polypeptide 23 (CYP2J23)
	Cytochrome P450, family 3 subfamily A, polypeptide 65 (CYP3A65)
	Cytochrome P450, family 46 subfamily A, polypeptide 61 (CYP46A1)
	Glutamate dehydrogenase 1a
	Glutathione peroxidase 1a
	Retinol saturase (all-trans-retinol 13,14-reductase)
	SH3 domain binding glutamic acid-rich protein like 3
	Sulfide quinone reductase-like
	Transferrin-a, Rho-class gluthatione S-transferase
Annotation Cluster 2	Pi-class glutathione-S-transferase
(Enrichment Score: 1.53)	Glutathione peroxidase 1a
	SH3 domain binding glutamic acid-rich protein like 3
	Transferrin receptor 1a
	Transferrin -a: Rho-class glutathione-S-transferase

**Table 5:** Functional Annotation Enrichment analysis of statistically-significant under-expressed genes in European eel silver males from the river Tiber site (N = 8) in comparison with silver males from the lake Bolsena site (N = 7). FE = Fold Enrichment

**Category**	**Term**	**Count**	**PValue**	**FE**
KEGG_PATHWAY	dre00190:Oxidative phosphorylation	32	<0.001	3.2
	dre04540:Gap junction	8	0.003	3.8
	dre03020:RNA polymerase	5	0.035	3.7
INTERPRO	IPR003008:Tubulin/FtsZ, GTPase domain	7	<0.001	7.9
	IPR017975:Tubulin, conserved site	7	<0.001	7.9
	IPR000217:Tubulin	7	<0.001	7.9
	IPR018316:Tubulin/FtsZ, 2-layer sandwich domain	7	<0.001	7.9
	IPR004217:Mitochondrial inner membrane translocase complex, Tim8/9/10/13-zinc finger-like	4	0.005	9.0
	IPR004217:Zinc finger, Tim10/DDP-type	4	0.005	9.0
	IPR002453:Beta tubulin	4	0.005	9.0
	IPR008011:Complex 1 LYR protein	4	0.012	7.2
	IPR013838:Beta tubulin, autoregulation binding site	4	0.012	7.2
	IPR003439:ABC transporter-like	5	0.018	4.5
	IPR001648:Ribosomal protein S18	3	0.034	9.0
	IPR017900:4Fe-4S ferredoxin, iron-sulphur binding, conserved site	3	0.034	9.0
	IPR012677:Nucleotide-binding, alpha-beta plait	11	0.034	2.1
	IPR003593:ATPase, AAA + type, core	8	0.042	2.4
SP_PIR	ribosomal protein	29	<0.001	3.2
	ribonucleoprotein	25	<0.001	3.5
	mitochondrion	21	<0.001	3.1
	ubiquinone	10	<0.001	4.0
	transit peptide	10	<0.001	3.6
	ribosome biogenesis	5	0.003	6.9
	translocation	5	0.003	6.9
	viral nucleoprotein	6	0.005	4.9
	mitochondrion inner membrane	9	0.005	3.1
	microtubule	7	0.009	3.6
	chaperone	9	0.011	2.8
	protein transport	8	0.016	2.9
	iron-sulfur	4	0.027	5.5
	iron	11	0.039	2.0
PIR_SUPERFAMILY	PIRSF002306:tubulin	7	<0.001	8.5
GOTERM_MF	GO:0005198 ~ structural molecule activity	41	<0.001	2.7
	GO:0003735 ~ structural constituent of ribosome	25	<0.001	2.6
	GO:0015078 ~ hydrogen ion transmembrane transporter activity	13	0.002	2.7
	GO:0015077 ~ monovalent inorganic cation transmembrane transporter activity	14	0.002	2.5
	GO:0009055 ~ electron carrier activity	15	0.001	2.6
	GO:0016887 ~ ATPase activity	15	0.006	2.2
	GO:0003723 ~ RNA binding	18	0.007	2.0
	GO:0022890 ~ inorganic cation transmembrane transporter activity	14	0.008	2.2
	GO:0015399 ~ primary active transmembrane transporter activity	9	0.009	2.9
	GO:0015405 ~ P-P-bond-hydrolysis-driven transmembrane transporter activity	9	0.009	2.9
	GO:0051536 ~ iron-sulfur cluster binding	6	0.012	4.0
	GO:0051540 ~ metal cluster binding	6	0.012	4.0
	GO:0016651 ~ oxidoreductase activity, acting on NADH or NADPH	7	0.020	3.1
	GO:0046961 ~ proton-transporting ATPase activity, rotational mechanism	5	0.024	4.2
	GO:0051537 ~ 2 iron, 2 sulfur cluster binding	3	0.032	9.2
	GO:0003899 ~ DNA-directed RNA polymerase activity	5	0.032	3.9
	GO:0034062 ~ RNA polymerase activity	5	0.032	3.9
	GO:0008135 ~ translation factor activity, nucleic acid binding	9	0.032	2.3
	GO:0003743 ~ translation initiation factor activity	7	0.037	2.7
	GO:0003924 ~ GTPase activity	7	0.037	2.7
GOTERM_CC	GO:0005739 ~ mitochondrion	43	<0.001	2.6
	GO:0030529 ~ ribonucleoprotein complex	46	<0.001	2.3
	GO:0044429 ~ mitochondrial part	31	<0.001	3.0
	GO:0005740 ~ mitochondrial envelope	24	<0.001	2.8
	GO:0005840 ~ ribosome	34	<0.001	2.2
	GO:0043228 ~ non-membrane-bounded organelle	61	<0.001	1.6
	GO:0043232 ~ intracellular non-membrane-bounded organelle	61	<0.001	1.6
	GO:0005743 ~ mitochondrial inner membrane	19	<0.001	2.8
	GO:0019866 ~ organelle inner membrane	19	<0.001	2.7
	GO:0031966 ~ mitochondrial membrane	20	<0.001	2.6
	GO:0031967 ~ organelle envelope	24	<0.001	2.3
	GO:0031975 ~ envelope	24	<0.001	2.3
	GO:0031090 ~ organelle membrane	24	0.001	1.9
	GO:0031974 ~ membrane-enclosed lumen	22	0.002	1.9
	GO:0005758 ~ mitochondrial intermembrane space	6	0.005	4.5
	GO:0031970 ~ organelle envelope lumen	6	0.005	4.5
	GO:0015934 ~ large ribosomal subunit	6	0.009	4.1
	GO:0042719 ~ mitochondrial intermembrane space protein transporter complex	4	0.011	6.8
	GO:0031980 ~ mitochondrial lumen	6	0.014	3.7
	GO:0005759 ~ mitochondrial matrix	6	0.014	3.7
	GO:0044455 ~ mitochondrial membrane part	6	0.021	3.4
	GO:0033279 ~ ribosomal subunit	7	0.037	2.6
	GO:0005874 ~ microtubule	7	0.037	2.6
GOTERM_BP	GO:0006412 ~ translation	36	<0.001	2.5
	GO:0042254 ~ ribosome biogenesis	10	<0.001	5.1
	GO:0022613 ~ ribonucleoprotein complex biogenesis	10	<0.001	4.6
	GO:0051258 ~ protein polymerization	7	<0.001	5.9
	GO:0070585 ~ protein localization in mitochondrion	5	<0.001	9.2
	GO:0006626 ~ protein targeting to mitochondrion	5	<0.001	9.2
	GO:0007005 ~ mitochondrion organization	5	<0.001	9.2
	GO:0006839 ~ mitochondrial transport	5	0.002	7.7
	GO:0055085 ~ transmembrane transport	20	0.002	2.1
	GO:0007006 ~ mitochondrial membrane organization	4	0.005	9.2
	GO:0045039 ~ protein import into mitochondrial inner membrane	4	0.005	9.2
	GO:0007007 ~ inner mitochondrial membrane organization	4	0.005	9.2
	GO:0007018 ~ microtubule-based movement	7	0.005	4.0
	GO:0006091 ~ generation of precursor metabolites and energy	16	0.005	2.1
	GO:0007017 ~ microtubule-based process	8	0.011	3.1
	GO:0022900 ~ electron transport chain	8	0.011	3.1
	GO:0034660 ~ ncRNA metabolic process	9	0.012	2.7
	GO:0006364 ~ rRNA processing	5	0.016	4.6
	GO:0016072 ~ rRNA metabolic process	5	0.016	4.6
	GO:0034622 ~ cellular macromolecular complex assembly	11	0.021	2.2
	GO:0006886 ~ intracellular protein transport	11	0.029	2.1
	GO:0034613 ~ cellular protein localization	11	0.029	2.1
	GO:0034621 ~ cellular macromolecular complex subunit organization	11	0.029	2.1
	GO:0006612 ~ protein targeting to membrane	4	0.031	5.3
	GO:0065002 ~ intracellular protein transmembrane transport	3	0.032	9.2
	GO:0070727 ~ cellular macromolecule localization	11	0.032	2.1
	GO:0046907 ~ intracellular transport	13	0.037	1.9
	GO:0034470 ~ ncRNA processing	6	0.037	3.1
	GO:0065003 ~ macromolecular complex assembly	12	0.037	1.9
	GO:0043623 ~ cellular protein complex assembly	7	0.038	2.7
	GO:0015985 ~ energy coupled proton transport, down electrochemical gradient	7	0.038	2.7
	GO:0034220 ~ ion transmembrane transport	7	0.038	2.7
	GO:0015986 ~ ATP synthesis coupled proton transport	7	0.038	2.7
	GO:0017038 ~ protein import	5	0.043	3.5
	GO:0043933 ~ macromolecular complex subunit organization	12	0.047	1.9
UP_SEQ_FEATURE	transit peptide:Mitochondrion	10	0.002	3.2
	short sequence motif:Twin CX3C motif	3	0.036	8.6
SMART	SM00382:AAA	8	0.009	3.2
	SM00360:RRM	10	0.012	2.6
	SM00651:Sm	4	0.042	4.8
COG_ONTOLOGY	Translation, ribosomal structure and biogenesis	7	0.015	3.1

**Table 6:** Functional Annotation Clustering of statistically-significant under-expressed genes in European eel silver males from the river Tiber site (N = 8) in comparison with silver males from the lake Bolsena site (N = 7)

**Annotation Cluster**	**Gene**
Annotation Cluster 1	Actin alpha 1, skeletal muscle
(Enrichment Score: 6.78)	Actin-related protein 2/3 complex, subunit 3
	Chromodomain helicase DNA binding protein 2
	Collagen, type I, alpha 1
	Collagen, type I, alpha 2
	DEAD (Asp-Glu-Ala-Asp) box polypeptide 51
	Dynein light chain 2
	Eukaryotic translation initiation factor 2 subunit 1 alpha
	Eukaryotic translation initiation factor 3 subunit 2 beta
	Eukaryotic translation initiation factor 3 subunit J
	Heterogeneous nuclear ribonucleoprotein D-like
	Isoleucyl-tRNA synthetase
	Keratin 8
	Mitochondrial ribosomal protein 63
	Mitochondrial ribosomal protein L13
	Mitochondrial ribosomal protein L15
	Mitochondrial ribosomal protein L17
	Mitochondrial ribosomal protein L23
	Mitochondrial ribosomal protein L38
	Mitochondrial ribosomal protein L45
	Mitochondrial ribosomal protein S18B
	Mitochondrial ribosomal protein S18C
	Mitochondrial ribosomal protein S23
	Mki67 (FHA domain) interacting nuclear phosphoprotein
	Profilin 2
	Ribosomal protein S8
	Ribosomal protein S29
	SDA1 domain containing 1
	Seryl-tRNA synthethase
	Slow myosin heavy chain 2
	Small nuclear ribonucleoprotein D1 polypeptide
	Small nuclear ribonucleoprotein D3 polypeptide
	Small nuclear ribonucleoprotein polypeptide E
	Small nuclear ribonucleoprotein polypeptide F-like
	Thrombospondin 1
	Thrombospondin 4b
	Titin a
	Tubulin, alpha 8 like 2
	Tubulin, alpha 8 like 4
	Tubulin, beta 5
	Vessicle-associated membrane protein, associated protein B and C
Annotation Cluster 2	ATP synthase, H+ transporting, mitochondrial F0 complex, subunit b
(Enrichment Score: 4.69)	ATP synthase, H+ transporting, mitochondrial F0 complex, subunit e
	ATP synthase, H+ transporting, mitochondrial F0 complex, alpha subunit 1
	ATPase, H+ transporting, lysosomal, V1 subunit H
	NADH dehydrogenase (ubiquinone) 1 beta subcomplex 4
	NADH dehydrogenase (ubiquinone) Fe-S protein 4
	Chromatin modifying protein 5
	COX17 cytochrome c oxidase assembly homolog
	Cytochrome c oxidase subunit VIIa 2
	Frataxin
	Heat shock protein 9
	Mitochondrial ribosomal protein 63
	Mitochondrial ribosomal protein L15
	Mitochondrial ribosomal protein L23
	Mitochondrial ribosomal protein L45
	Nuclear cap binding protein subunit 2
	SDA1 domain containing 1
	Solute carrier family 25, member 16
	Succinate dehydrogenase complex, subunit A, flavoprotein
	Succinate dehydrogenase complex, subunit B, iron sulfur
	Succinate dehydrogenase complex, subunit D, integral membrane protein A
	Succinate dehydrogenase complex, subunit D, integral membrane protein B
	Succinate dehydrogenase complex, subunit A, flavoprotein
	Transcription factor B2
	Ubiquinol-cytochrome c reductase, Rieske iron-sulfur polypeptide 1

When repeating the analysis with females from Bolsena also included (total N = 23 individuals), 1,135 transcripts were differentially expressed in the Tiber samples in comparison with the Bolsena samples using a 5% FDR, 552 transcripts over-expressed and 583 transcripts under-expressed. Enrichment analysis using DAVID confirmed the results obtained with the males dataset and all key genes over-expressed (genes involved in drug metabolism) and under-expressed (genes involved in energy metabolism) in the individuals from Tiber were also significantly enriched when females were added to the analysis. Unlike gene expression profiles, which were strongly affected by sexes, enrichment analyses did not vary when including females, which suggests that the differentially expressed genes between Tiber and Bolsena were under-represented among the sub-set of 2,000 genes used in the Pearson correlation-based heatmap.

When correlating gene expression and individual measures of pollutants in SAM, a total of 14 transcripts were positively correlated (1 with PCB and the rest with metals) and a total of 27 transcripts were negatively correlated (2 with PCB, 7 with OCPs, 13 with BFRs and 5 with metals). In both cases, the same associations were obtained using either a 0, 1 or 5% FDR. Using the list of positively and negatively correlated transcripts, a putative match with zebrafish was found for 20 transcripts to be used in DAVID. A total of 11 terms were enriched (all negatively correlated with individual measures of pollutants), but only one term was statistically significant (p = 0.015; Table 7), corresponding to NADH-ubiquinone oxidoreductase chain 2 and 5 genes. No functional annotation clusters or KEGG pathways were enriched.

**Table 7:** Functional Annotation Genes correlated with pollutants at the individual level. FE = Fold Enrichment

**Category**	**Term**	**Count**	**P value**	**FE**
INTERPRO	IPR002750: NADH	2	0.015	122.7
KEGG_PATHWAY	dre03010: Ribosome	3	0.072	5.8
SP_PIR_KEYWORDS	respiratory chain	2	0.065	27.5
	electron transport	2	0.085	20.6
GOTERM_BP	GO:0042773 ATP synthesis coupled electron transport	2	0.054	32.9
	GO:0022904 respiratory electron transport chain	2	0.072	24.7
GOTERM_MF	GO:0050136 NADH dehydrogenase (quinone) activity	2	0.054	32.9
	GO:0008137 NADH dehydrogenase (ubiquinone) activity	2	0.054	32.9
	GO:0016655 Oxireductose (NADH) activity	2	0.054	32.9
	GO:0003954 NADH dehydrogenase activity	2	0.054	32.9
	GO:0003735 Structural component of ribosome	2	0.042	8.7

### Estimation of evolutionary distance of CYP3A genes

**Figure 7:**
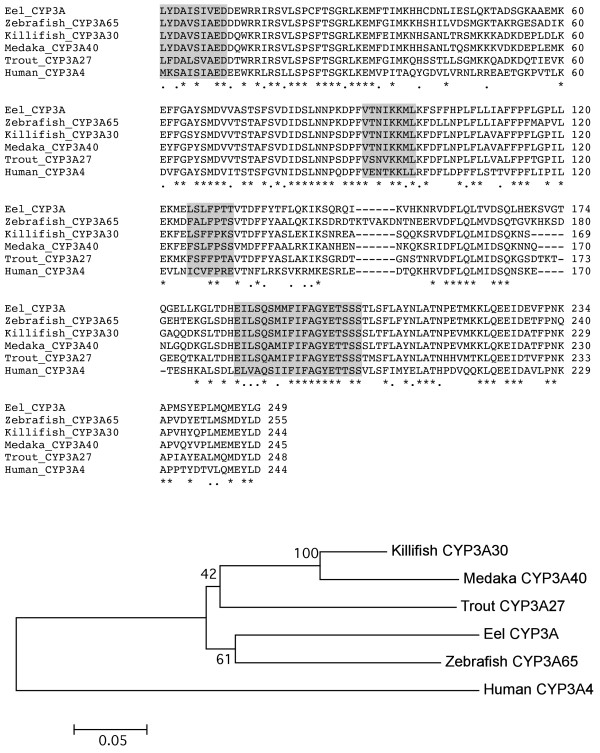
**Alignment of the amino acid sequence of European eel CYP3A and other vertebrate CYP3A members with high amino acid sequence similarity using the CLUSTAL_W method.** Amino acids shared by all species are indicated by a stark mark and amino acids shared by all fish species by a dot mark. Putative recognition sites SRS are indicated by light shade. A gene tree was constructed using the Neighbour-Joining tree method in MEGA5. Genbank accession numbers for the sequences used are as follows: zebrafish CYP3A65, AY452279; killifish CYP3A330, AF105068; medaka CYP3A40, AF251271; rainbow trout CYP3A27, U96077; human CYP3A4, NP059488.

Our study shows one CYP3A isozyme over-expressed in the Tiber samples, with a positive BLAST hit with zebrafish *Danio rerio* CYP3A65 [40], one of the key enzymes involved in the detoxification of xenobiotic substances. Estimation of evolutionary distances using pairwise distance (p-distance model) in MEGA 5 [41] showed a genetic distance of 0.308 between the European eel CYP3A and zebrafish CYP3A65. Comparison with other amino acid sequences available in GenBank showed slightly higher pairwise genetic distances between the European eel CYP3A and killifish *Fundulus heteroclitus* CYP3A30 (0.321), medaka *Oryzias latipes* CYP3A40 (0.365) and rainbow trout *Oncorhynchus mykiss* CYP3A27 (0.372). By comparison, a higher genetic distance was found between the European eel CYP3A and the human CYP3A4 of 0.614. A Neighbour-Joining tree comparing those CYP3A isozymes grouped together the European eel and zebrafish CYP3As, separated from the killifish, medaka and rainbow trout, which formed a second group (Figure 7), while the human CYP3A4 appeared as the most distant.

## Discussion

### Over-expression of detoxification genes

Several genes related to metabolism of xenobiotics were upregulated in the highly polluted site (Tiber) in comparison with the low pollution site (Bolsena), which indicates that their expression is pollutant-related. These include a set of cytochrome P450 (CYP) genes that were upregulated by 8–10 fold in the Tiber sample group. The liver CYP enzymes are a superfamily of heme-containing monooxygenases that constitute the principal enzymes involved in metabolism of xenobiotics. The function of most CYPs is to catalyze the oxidative and reductive metabolism of xenobiotic substances such as drugs, environmental chemicals and endogenous substrates [42]. Out of all families, CYP3A isozymes comprise the largest proportion of CYPs in the liver. CYP3A isozymes are involved in the first phase of the xenobiotic metabolism by means of introducing reactive or polar groups into the xenobiotics, thus modifying their chemical structure. Previous studies have shown that expression of CYP3A isozymes is induced by dioxins [40], PCBs [43] and PBDEs [44].

A novel European eel CYP3A gene, upregulated in the highly polluted Tiber site, was identified by enrichment analysis and shown to be ortholog to zebrafish *Danio rerio* CYP3A65 by phylogenetic analysis. The European eel CYP3A conformed to the specific structural features associated with the family, and despite being a partial sequence, several conserved regions could be identified including a total of four substrate recognition sites (partial SRS1, full SRS2, SRS3 and SRS4). Differences in CYP catalytic activities are suggested to be determined by amino acid changes in SRS sites [45]. In the case of the European eel CYP3A, differences in amino acid sequence between the species in Figure 7 were predominantly observed in SRS3 (43%) but not in SRS2 (0%) or SRS4 (6%), which agrees with the observations when comparing two CYP3A paralogs in medaka [45].

In addition to xenobiotic metabolizing enzymes involved in the first phase of detoxification, we also identified genes involved in the second phase of the xenobiotic metabolism, particularly glutathione-S-transferase (GST), which was over-expressed by 1.5 fold in the heavily-polluted Tiber site. Following addition of reactive functional groups by CYPs in phase I, phase II enzymes catalyze the conjugation of the xenobiotic parent compound or its metabolites with an endogenous ligand, thus facilitating its excretion [46]. GSTs are a multigene superfamily of dimeric multifunctional enzymes that catalyze the conjugation of metabolites by covalently adding reduced glutathione (GSH). Based on their biochemical, immunologic and structural properties, GSTs are categorized into 4 main classes: alpha, mu, pi, and theta. The gene over-expressed in the Tiber samples was glutathione S-transferase pi (GSTP1), previously described in many fish species including the European eel (GenBank accession number AAS01601). In contrast with phase I enzymes, an increase in GST activity as a response to xenobiotics like the one found in our study in the highly polluted Tiber site does not seem to be universal, and while some studies show a higher enzyme activity, others do not observe changes or even report negative results. In this sense, a review of 43 laboratory and 39 field studies in fish only reported a significant GST increase in 33% of the studies [46]. Besides biotransformation or metabolic enzymes, many pollutants have been shown to exert toxic effects related to oxidative stress [47]. Antioxidant enzymes such as superoxide dismutase (SOD), catalase (CAT) or glutathione peroxidase (GPOX) defend the organisms against oxygen toxicity and are critical during the detoxification of radicals to non-reactive molecules. In our study, GPOX was upregulated in samples from the highly-polluted Tiber site and was included in the two annotated clusters that were significantly enriched. GPOX is a peroxidase that plays an important role in protecting membranes from lipid peroxidation damage. The increased GPOX activity observed in our study contrasts with the lack of response of GPOX to pollution found in most studies. The review of Van der Oost et al. [46] reported a positive response on GPOX in only 14% of field studies, limited to chub *Squalius cephalus* and grey mullet *Mugil cephalus*. Regarding other antioxidant enzymes, the European eel platform developed in this study included 3 transcripts encoding for SOD, only one of which was upregulated in the samples from the Tiber site, although not significantly so. No transcripts showing a significant match with CAT were included in the platform.

Along with detoxification genes, environmental stress has been proposed to induce the synthesis of proteins involved in protection and repair of the cell in response to environmental perturbations, including stress (heat shock) proteins, metallothioneins and multi-xenobiotic resistance (MXR) transmembrane proteins. In particular, metallothioneins constitute a superfamily of proteins involved in the regulation and/or detoxification of a wide variety of metals (e.g., Cd, Cu, Zn, Hg, Co, Ni, Bi and Ag; [48,49]). However, in our study, metallothioneins were not over-expressed in samples from the highly-polluted Tiber site. The European eel array developed in this study included at least 11 transcripts encoding metallothioneins, and while most of them (82%) showed a higher expression in Tiber than in Bolsena samples, differences were not statistically significant. In contrast with our study, a straightforward positive dose–response association to single pollutants for metallothioneins has been documented in experimental studies in European glass eels [32]. However, many biotic and abiotic factors can influence gene expression in nature [10], leading to discrepancies between field and experimental studies [50]. On the other hand, examination of differences in gene expression between polluted and unpolluted populations of Atlantic cod (*Gadus morhua*) from western Norway using gene expression profiling showed genes related to metal stress including metallothionein to be upregulated in the polluted locations [51], which confirms early experimental results in fish (i.e. brown trout, [52]). The recent study of Milan et al. [53] on expression profiling in the Manila clam *Ruditapes philippinarum* reported four transcripts encoding metallothioneins over-expressed in samples from a highly polluted area. In the same study, up to nine GST-coding transcripts were upregulated in the polluted area.

Some of the genes over-expressed in the highly polluted Tiber site could be potentially used as genomic markers (biomarkers) for environmental genomics, including the European eel CYP3A gene ortholog to CYP3A65 in zebrafish, glutathione-S-transferase and glutathione peroxidase. However, it should be noted that further studies are required to confirm the adequacy of the potential biomarkers identified in our study, including a full characterization of the genes, the analysis of variation across sexes and habitats and the temporal stability of their expression, in order for them to be unequivocally used as valid biomarkers.

### Pollution is associated with decreased energy production

Our results suggest that, together with the induction of detoxification genes, pollution is associated with decreased respiratory activity and energy production. Many genes involved in energy metabolism were downregulated in samples from the highly-polluted Tiber site, including key enzymes in the mitochondrial respiratory chain and oxidative phosphorylation (Figure 4), and were grouped in a significantly enriched annotation cluster (enrichment score of 4.69). The mitochondrial respiratory chain couples electron transfer between electron donors (NADH, succinate) and acceptors (O_2_) with the transport of protons (H^+^ ions) from the mitochondrial matrix into the intermembrane space, which builds a proton gradient across the mitochondrial inner membrane that is used to generate energy in the form of ATP. The following genes were all down-regulated in tissues collected from the Tiber fish:

(1) NADH dehydrogenase. This enzyme is a component of Complex I of the mitochondrial respiratory chain that catalyzes the transfer of electrons from NADH to coenzyme Q (ubiquinone).

(2) Succinate dehydrogenase. This enzyme is part of Complex II of the mitochondrial respiratory chain that catalyzes the conversion from succinate to fumarate and passes electrons to coenzyme Q in the same fashion as NADH.

(3) Ubiquinol-cytochrome *c* reductase. This enzyme is part of the third complex in the electron transport chain, which catalyzes the oxidation of coenzyme Q. Electrons are sequentially transferred from coenzyme Q to complex III, and from there to cytochrome *c*, a water-soluble electron carrier located within the intermembrane space.

(4) Cytochrome *c* oxidase. This enzyme is part of Complex IV of the mitochondrial respiratory chain that receives electrons from cytochrome *c* and transfers them to oxygen, the most electronegative and terminal electron acceptor in the chain, converting molecular oxygen to two molecules of water. In the process, it translocates four protons across the membrane, establishing a transmembrane proton gradient used by the ATP synthase to synthesize energy.

(5) ATP synthase. This enzyme is sometimes referred as Complex V of the electron transport chain. The creation of a proton gradient across the mitochondrial inner membrane is used by ATP synthase to synthesize ATP via oxidative phosphorylation, using the flow of H^+^ to phosphorylate ADP to ATP.

In agreement with our study, experimental studies documented a decrease in expression of genes involved in the respiratory chain in response to cadmium exposure and hypoxia in the gills of glass eels [32].

In addition to genes involved in energy metabolism, proteins from two other gene families were downregulated in samples from the highly-polluted Tiber site, namely gap junction (Figure 5) and RNA polymerase (Figure 6) genes. Gap junctions are channels that connect the cytoplasms of adjacent cells, allowing ions and small solutes to pass between them. Among the postulated roles of gap junction communication are coordination of activities of specific groups of cells, nutrient sharing, regulation of growth and oncogenesis [54], although a connection with pollution does not seem straightforward. On the other hand, the multi-subunited RNA polymerases are the main enzymes responsible for gene transcription, which is the process of creating a complementary RNA copy of a DNA sequence. Enriched nodes in our study represented subunits of RNA polymerase II, which catalyzes the transcription of DNA to synthesize precursors of messenger RNA (mRNA) but also some non-coding RNAs. RNA expression is frequently used as a proxy of condition [55], as the amount of ribosomal, messenger and transfer RNAs provides information on the metabolic status of the whole organism. Thus, the suggested lower RNA expression in samples from the highly-polluted site points to a lower metabolic rate in this group of fish.

## Conclusions

Our findings suggest that pollution affects the condition of eels from a metabolic point of view, seeing that key genes involved in respiratory activity, energy production and RNA expression are downregulated in the Tiber fish, possibly resulting in a low energetic status of the individuals. Although we did not measure metabolism directly, the suggested genome-wide lowered metabolic rate and condition observed in eels at the pre-migrating stage in our study points to a poor quality of spawners that could potentially impair both spawning migration and reproduction in the Sargasso Sea. Given that many river basins across Europe are experiencing similar levels of environmental pollution, an impoverished spawning stock could lead to a reduced reproductive success, a lowered effective population size and an impaired evolutionary potential.

## Methods

### Contigs assembly and transcripts annotation

A total of 640,040 sequence reads from various existing and novel sources were assembled into contigs, including: (1) 242,762 reads obtained by 454 FLX Titanium sequencing of a normalised cDNA library produced from a pool of 18 glass eels (cephalic region) that constituted the first Eeelbase sequencing run [56]; (2) 300,555 additional reads from a second Eeelbase sequencing run using the same cDNA library; (3) 93,119 reads also obtained by 454 sequencing of a cDNA library produced from olfactory gland of adults (A. Canario, unpublished data) and (4) 3,604 reads obtained by Sanger sequencing using a mixture of adult tissues (brain, gill, intestine and kidney) [37].

Sequence reads were assembled into contigs using MIRA 3 assembler [57]. As in Coppe et al. [56] we used a double assembly approach, with the first run of hybrid assembly being used for a second local assembly to reduce contig redundancy. Threshold values were then applied to obtain the final set of contigs representing European eel transcripts: a minimum contig length of 200 nucleotides and a minimum average sequence quality of 30 (Phred Scores), corresponding to an error rate of 1 per 1,000 bp.

*De novo* functional annotation of the European eel transcriptome was obtained by similarity using BLAST (Basic Local Alignment Search Tool) and Blast2Go [58]. Batch BLAST similarity searches for the full transcriptome were conducted locally against both NCBI (National Centre for Biotechnology Information) nucleotide (nt) and protein non-redundant (nr) databases using BLASTN and BLASTX, respectively. Alignments with an e-value smaller than 1E^-3^ were considered. The Blast2Go suite was used for functional annotation of transcripts, mapping GO (Gene Ontology) terms to those transcripts with BLAST hits. In order to provide a broader overview of the ontology content, GO classes were grouped into GO-slim terms, which are cut-down versions of the GO ontologies containing a subset of GO terms, using the web tool CateGOrizer (http://www.animalgenome.org/bioinfo/tools/countgo/). The provisional annotation for microarray development was performed in Spring 2011, when the recently published eel draft genome [59] was not available.

### DNA microarray design

Probe design started with selection of target sequences to be included in the European eel microarray. We included all annotated sequences similar to vertebrates and invertebrates hits. Bacteria, virus, plants, fungi and protozoa were excluded. For 12,296 contigs with BLASTX hits, the putative orientation was unambiguous across hits and a single sense probe was designed. For 1,390 contigs, most with BLASTX hits and few with BLASTN hits, the putative orientation was ambiguous and two probes with opposite orientation (sense and antisense) were designed. In order to minimize assembler redundancy, 163 contigs differing by less than 3 nucleotide changes were discarded using a custom-made script to find best BLAST matches within the contig database. The final number of probes was 14,913. Probe design was carried out using the Agilent eArray Interface (https://earray.chem.agilent.com/earray/), which applies proprietary prediction algorithms to design 60-mer oligoprobes. Microarrays were synthesized in situ using the Agilent ink-jet technology with an 8 x 15 K format (8 arrays in a single slide). Each array included default positive and negative controls. Probe sequences and other details on the microarray platform can be found in the GEO database under accession number GPL15124.

### Sample collection

Samples of European eel were collected by fishermen during October 2009 in two separate areas in the Lazio region (Central Italy) characterized by different levels of environmental pollutants: the low course of the river Tiber (41°48′N; 12°25′E) and the nearby lake Bolsena (42°36′N; 11°55′E).

The river Tiber drains the largest basin in central Italy, flowing through a highly urbanised area around the city of Rome before flowing into the Tyrrhenian Sea. Our sampling area is situated 20 km from the river mouth on the lower river stretches, downstream from Rome, and shows a salinity of 0 ‰. This section of the river contains the highest pollution level as it collects many sources of organic and inorganic contaminants, including runoffs from intensive agriculture and industry outlets, the heavily polluted Aniene river tributary and the city sewage outlets. Although the city runoffs are properly treated, the river still receives quantities of incompletely treated wastewater and sewage from the urban area [60,61]. On the other hand, Bolsena is a freshwater (salinity of 0 ‰) mid-sized lake of volcanic origin, with a 113.6 km^2^ surface, a maximum length of 13.5 km and a mean depth of 81 m. The lake receives most of its water from rainfall as the hydrographic network of this area contains few waterways. Its only effluent is the river Marta, which connects the lake Bolsena to the Tyrrhenian Sea. Overall, the lake exhibits a good environmental quality despite a recent increase in tourism, urbanism and agriculture use. The lake is characterized by low residues of organochlorine pesticides and PCBs, well below the Italian and European action levels [62].

All individuals were silver eels (adults) as determined by gonad development stage and computed Pankhurst's [63] ocular index (OI), which reflects changes in eye diameter during metamorphosis to the silver stage. At the river Tiber, all samples were males (N = 30), measuring 35–42 cm total length (TL) and 81–144 g body weight (BW). At lake Bolsena, samples were constituted by a mix of males (N = 9; 37–42 cm TL, 77–121 g BW) and females (N = 21; 62–92 cm TL; 358–1,333 g BW). Individuals were sacrificed immediately after collection from the field following international recognized guidelines and according to National laws in order to minimize pain and stress. Biopsies of muscle and liver were collected for pollutants (stored at −20°C) and microarray (stored in RNALater) analyses, respectively. No analyses or experiments were conducted with live animals. The University of Padova ethic board CEASA (Comitato Etico di Ateneo per la Sperimentazione Animale) exempted this study from review as an extra moenia (in the field) activity.

### Pollutants measurements and analysis

A total of 36 males were analysed for PCBs, several organochlorine pesticides (OCPs) and brominated flame retardants (BFR) and nine metals. The analysis included 6 males from the low-pollution site at lake Bolsena, plus a higher number of males (N = 30) from the heavily polluted river Tiber site. From each individual, several samples of muscle tissue from the central part of the body (10 g wet weight each; ww) were removed in order to conduct all the measurements for pollutants.

Samples were analysed for 36 different PCB congeners, including the seven congeners considered as indicator PCBs (IUPAC No. 28, 52, 101, 118, 138, 153 and 180). OCPs in this study included hexachlorobenzene (HCB), pp-DDT and its major metabolite pp-DDE. BFRs included ten PBDE congeners (No. 28, 47, 49, 66, 85, 99, 100, 153, 154 and 183) and three HBCD isomers. Finally, the levels of nine metals were determined: silver (Ag), arsenic (As), cadmium (Cd), cobalt (Co), chromium (Cr), copper (Cu), nickel (Ni), lead (Pb) and zinc (Zn).

About 1 g eel muscle tissue was homogenised with Na_2_SO_4_, spiked with internal standards (BDE 77, BDE 128, ^13^C-BDE 209, ^13^C-α-HBCD, ^13^C-β-HBCD and ^13^C-γ-HBCD), extracted by hot Soxhlet during 2 h with hexane:acetone (3:1) and cleaned-up on acidified silica [64]. Prior to the clean-up, a fraction of the extract was taken to determine the lipid content gravimetrically. After clean-up and fractionation, PCBs, pp-DDE, pp-DDT and HCB were analysed by Gas chromatography–mass spectrometry (GC-MS) with Electron Impact Ionization (EI), PBDEs by GC-MS with Electron-capture Negative Ionization (ECNI) and HBCDs by Liquid Chromatography- Mass Spectrometry (LC-MS). Details are described in Roosens et al. [64]. The quality control procedures included the analysis of procedural blanks, duplicate samples, and certified material SRM 1945. Obtained values were deviating <10% from the certified values and all samples were blank-corrected. Recoveries of internal standards were all above 80%, RSD < 10%. Method quantification limits (LOQs) for individual pollutants were based on procedural blanks (10 x SD) and the amount of sample taken for analysis (typically 1 g eel muscle). LOQs ranged between 0.1 and 0.4 ng/g lipid wet weight.

Between 3 and 5 g of muscle tissue from the posterior part of the body was used for metal determination. Tissue samples were transferred to pre-weighted acid washed polypropylene vials and dried for 24 h at 60°C. Subsequently, the biological material was digested in a microwave oven, by adding a mixture (5:1) of nitric acid (70%) and peroxic acid (30%) [65]. Digested samples were frozen at −20°C until further analysis. Metals in muscle were analyzed using a High Resolution Inductively Coupled Plasma Mass Spectrometer (HR-ICP-MS). Concentrations of the metals in the muscle were calculated on a dry weight basis and expressed as μg/g. All samples were analysed in batches with blanks. Analytical accuracy was determined using certified reference material of the Community Bureau of Reference, i.e. standard for trace elements in mussel tissue (CRM 278). Recoveries were within 10% of the certified values.

Bioaccumulation of pollutants in individuals from the Tiber and Bolsena sites was compared with an univariate ANOVA for each pollutant separately. A Multivariate ANOVA (MANOVA) was conducted on a set of all pollutants combined (Sum 36 PCBs, HCB, pp-DDE, pp-DDT, Sum 10 PBDEs, Sum 3 HBCDs and nine metals). Significance for all statistic tests was taken as *p* = 0.05. All statistic analyses were performed in STATISTICA v. 10.0 (Statsoft).

### RNA extraction, labelling and hybridization

Gene expression analysis was carried out for a total of 23 individuals: 8 males from the highly polluted site at the river Tiber and 15 individuals from the low-pollution site at lake Bolsena, which included both males (N = 7) and females (N = 8). For each individual, total RNA was extracted from a 20–30 mg piece of liver using the RNAeasy Mini Kit (Qiagen). RNA concentration was determined using a Nanodrop ND-1000 spectrophotometer (NanoDrop Technologies). RNA integrity and quality was estimated on an Agilent 2100 Bioanalyzer (Agilent Technologies). Minimum RNA Integrity Number (RIN) was 7.5.

Sample labelling and hybridization were carried out following the Agilent One-Color Microarray-Based Gene Expression Analysis protocol (Low Input Quick Amp Labelling). For each individual, 100 ng total RNA were linearly amplified and labelled with the fluorescent dye Cy3-dCTP. In order to monitor microarray analysis work-flow, Agilent Spike-in Mix (a mixture of 10 different viral poly-adenylated RNAs) was added to each RNA sample before amplification and labelling. Labelled cRNA was purified with Qiagen RNAeasy Mini Kit and sample concentration and Cy3 specific activity were measured using a Nanodrop ND-1000 spectrophotometer. A Cy3 specific activity between 8 and 17 pmol Cy3 per μg cRNA was considered adequate for hybridization. Prior to hybridization, a total of 600 ng of labelled cRNA was fragmented for 30 min at 60°C by adding 5 μl 10X Blocking Agent and 1 μl Fragmentation buffer, and finally diluted with 25 μl 2X GE Hybridization buffer. A volume of 40 μl was dispended into the backing slide, assembled to the microarray slide (each slide containing eight arrays) and placed in the hybridization chamber. Slides were incubated for 17 h at 65°C in an Agilent Hybridization Oven. Afterwards, slides were removed from the hybridization chamber, disassembled in GE Wash Buffer 1, and washed for 1 min in GE Wash Buffer 1 followed by one additional wash for 1 min in GE Wash Buffer 2.

### Microarray analysis

Hybridized slides were scanned at 5 μm resolution using an Agilent DNA microarray scanner. Slides were scanned at two different sensitivity levels (XDR Hi 100% and XDR Lo 10%) and the two linked images generated were analysed together. Data were extracted and background subtracted using the standard procedure in Agilent Feature Extraction (FE) software v. 9.5.1. Hybridization success was evaluated using flag values, excluding those intensities not equal to 1. Normalization procedures were performed using the R statistical software (http://www.r-project.org), with Spike-In control intensities used to identify the best normalization procedure. Quantile normalization always yielded better results than cyclic loess normalization and quantile-normalized data were used in all subsequent analysis. Normalized fluorescent data have been deposited in the GEO database under accession number GSE35055.

A Pearson correlation-based heatmap representation of the gene expression profile of the individuals was drawn using an unsupervised clustering approach implemented in R. Cluster analysis was applied to the 2,000 genes with mean expression over a value of 100 and showing the most variable expression profiles across samples according to variation coefficient.

Differentially expressed genes across samples were identified using the statistical tests implemented in the program SAM (Significance Analysis of Microarrays) release 4.0 [66]. The two class unpaired test was used to identify over- and under-expressed genes between Tiber (heavily polluted) and Bolsena (lowly polluted) sites. A minimum of 1.5 fold change between groups was considered. The quantitative test was used to identify a correlation between gene expression and individual measure of PCBs, OCPs, BFRs and metals. We applied a 5% false discovery rate for multiple testing using the q-value method presented in Storey [67].

Functional annotation analysis of differentially expressed genes between Tiber and Bolsena sites was performed using the DAVID (Database for Annotation, Visualization and Integrated Discovery) web-server v6.7 (http://david.abcc.ncifcrf.gov). Prior to the analysis in DAVID, it was necessary to link differentially expressed sequences with sequence identifiers that could be recognized in DAVID. To do so, a BLAST search was conducted for significant matches of the European eel transcripts directly against zebrafish *Danio rerio* Ensembl proteins using BLASTX. *Danio rerio* Ensembl Gene IDs were obtained from the corresponding Ensembl protein entries using the Biomart data mining tool in the Ensembl website (http://www.ensembl.org/biomart/). Gene functional analysis in DAVID was conducted defining the zebrafish IDs corresponding to differentially expressed European eel transcripts as 'Gene list' and the zebrafish IDs corresponding to all genes represented in the array as 'Background'. Standard settings of gene count = 2 and ease = 0.1 were used. Additionally, a functional annotation clustering analysis was also conducted, in which genes were grouped into functionally related sets of genes. Minimum overall enrichment score for each group based on the EASE scores of each term members was set to 1.3, which is equivalent to the non-log scale of 0.05.

## Competing interests

The authors declare that they have no competing interests.

## Authors’ contributions

LZ and JMP conceived and designed the project, with substantial contributions from EC, GEM, MM and LB. SB and AC conceived and implemented the database and conducted all bioinformatic analyses together with JMP. CB, LB and AC conducted the analyses of pollutants. JMP, IAMM and LZ carried out the probe design. MM and IAMM carried out the microarray experiments. JMP and IAMM carried out the functional annotation analysis. JMP, LZ and GEM interpreted the data. JMP wrote the paper with contributions from GEM, LZ, SB, EC, FC, CB, LB, AC, GC, IAMM, MM, LB and TP. All authors read and approved the final version of the manuscript.
